# 3D printed anthropomorphic left ventricular myocardial phantom for nuclear medicine imaging applications

**DOI:** 10.1186/s40658-022-00461-3

**Published:** 2022-05-03

**Authors:** Janos Kiss, Laszlo Balkay, Kornel Kukuts, Marton Miko, Attila Forgacs, Gyorgy Trencsenyi, Aron K. Krizsan

**Affiliations:** 1 Division of Radiology and Imaging Science, Department of Medical Imaging, Faculty of Medicine, University of Debrecen, Nagyerdei krt. 98., Debrecen, 4032 Hungary; 2 Division of Nuclear Medicine and Translational Imaging, Department of Medical Imaging, Faculty of Medicine, University of Debrecen, Nagyerdei krt. 98., Debrecen, 4032 Hungary; 3 ScanoMed Nuclear Medicine Centers, Nagyerdei krt. 98., Debrecen, 4032 Hungary; 4 Mediso Ltd., Laborc Utca 3., Budapest, 1037 Hungary

**Keywords:** Cardiac, SPECT, 3D printing, Phantom, Technetium-99m

## Abstract

**Background:**

Anthropomorphic torso phantoms, including a cardiac insert, are frequently used to investigate the imaging performance of SPECT and PET systems. These phantom solutions are generally featuring a simple anatomical representation of the heart. 3D printing technology paves the way to create cardiac phantoms with more complex volume definition. This study aimed to describe how a fillable left ventricular myocardium (LVm) phantom can be manufactured using geometry extracted from a patient image.

**Methods:**

The LVm of a healthy subject was segmented from ^18^F-FDG attenuation corrected PET image set. Two types of phantoms were created and 3D printed using polyethylene terephthalate glycol (PETG) material: one representing the original healthy LVm, and the other mimicking myocardium with a perfusion defect. The accuracy of the LVm phantom production was investigated by high-resolution CT scanning of 3 identical replicas. ^99m^Tc SPECT acquisitions using local cardiac protocol were performed, without additional scattering media (“in air” measurements) for both phantom types. Furthermore, the healthy LVm phantom was inserted in the commercially available DataSpectrum Anthropomorphic Torso Phantom (“in torso” measurement) and measured with hot background and hot liver insert.

**Results:**

Phantoms were easy to fill without any air-bubbles or leakage, were found to be reproducible and fully compatible with the torso phantom. Seventeen segments polar map analysis of the "in air” measurements revealed that a significant deficit in the distribution appeared where it was expected. 59% of polar map segments had less than 5% deviation for the "in torso” and "in air” measurement comparison. Excluding the deficit area, neither comparison had more than a 12.4% deviation. All the three polar maps showed similar apex and apical region values for all configurations.

**Conclusions:**

Fillable anthropomorphic 3D printed phantom of LVm can be produced with high precision and reproducibility. The 3D printed LVm phantoms were found to be suitable for SPECT image quality tests during different imaging scenarios. The flexibility of the 3D printing process presented in this study provides scalable and anthropomorphic image quality phantoms in nuclear cardiology imaging.

## Background

Performance measurements and optimization of nuclear medicine imaging systems involve the use of different phantoms to mimic human activity distributions [1–3]. Accurate anthropomorphic phantoms have been introduced to reveal quantitative inaccuracies and to detect the presence of image artefacts caused by inappropriate acquisition, reconstruction, and image processing [4–8]. Several of these phantoms are commercially available, generally with fixed size and geometry. 3D printing technology including direct ink writing [9], fused deposition modelling (FDM) [10–13], digital light processing (DLP) [14] or stereo-lithography (SLA) [15] offers large variety of possibilities to design custom-made geometrical and anthropomorphic phantoms [16–23]. A systematic review by Filippou et al. concludes that 3D printing methods can complete or replace commercially available phantoms in the fields of CT, MRI, PET, SPECT, US, and mammography imaging [24]. Several studies reveal 3D printed phantom solutions for nuclear medicine applications using real patient imaging data, including fillable multicompartmental torso in quantitative imaging analysis for ^90^Y-DOTATATE radiopeptide therapy [25], fillable kidney phantom for ^177^Lu SPECT reconstruction optimization [26], as well as tumor phantom set of various shapes for testing comparison of PET radiomics features in a multi-center approach [27]. Focusing on cardiac phantom solutions, Matsutomo et al. designed and printed a set of specific inserts to simulate different ischemic levels to complete the commercially available Myocardial SPECT Phantom HL (Kyoto Kagaku Co., Ltd., Kyoto, Japan) [28]. Grice et al. introduced a left ventricle (LV) cardiac phantom with simplified wall geometry containing low perfusion lesions within a non-anthropomorphic background container, printed from polylactic acid (PLA) material [29]. The endeavor of creating new cardiac phantoms is encouraged by clinically relevant, but unanswered methodological questions. The lack of geometrically appropriate cardiac phantom prevents the investigation of image artefacts and image processing failures attributed to the inhomogeneity of the cardiac wall thickness. 3D printing technology makes it possible to create the complex geometry of a real heart, which is not feasible with traditional manufacturing methods. This study aimed to determine whether creating a fillable, anatomically accurate 3D printed left ventricle myocardium (LVm) phantom segmented from a PET image volume of a real patient is feasible. The suitability of polyethylene terephthalate glycol (PETG) plastic for anthropomorphic LVm phantom production is demonstrated for the first time. The phantom insert was designed to be compatible with the commercially available Anthropomorphic Torso Phantom (DataSpectrum Co., Durham, NC). CT images are presented to confirm the reproducibility of 3D printing and phantom preparation. Furthermore, SPECT measurements were performed to demonstrate that the proposed phantoms give a complementary solution to the currently available phantoms in the field of nuclear cardiology imaging.

## Materials and methods

### Phantom design

**Fig. 1:**
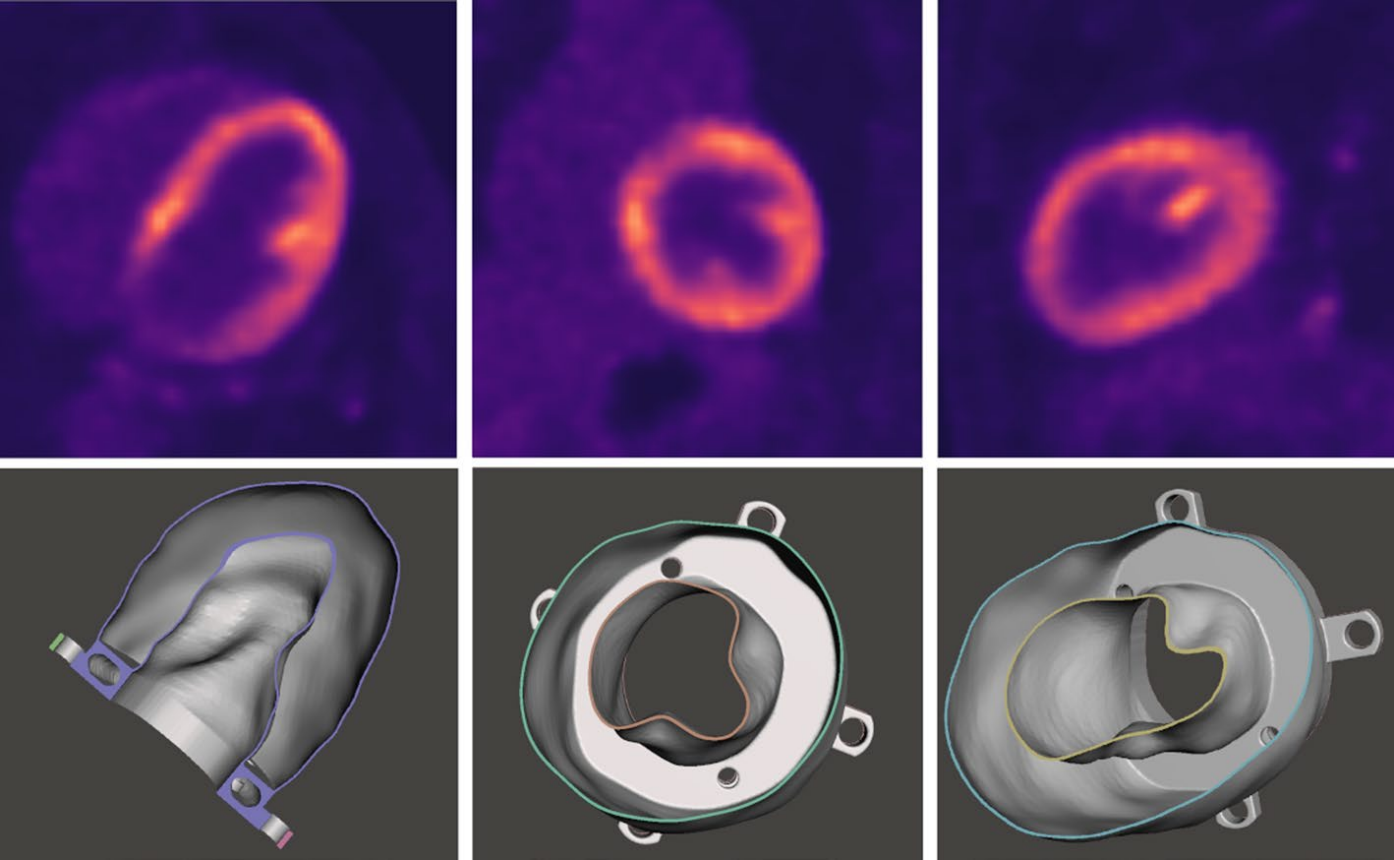
Three orthogonal views of reconstructed ^18^F-FDG PET/CT image (upper row) and the 3D phantom design (bottom row)

Input image data for the phantom design were extracted from an ^18^F-FDG PET/CT study of a patient (age: 67 years; weight: 63 kg) without known coronary artery disease. Whole body PET/CT acquisition was performed on a GE Discovery MI system, using the local patient examination protocol (injected dose 220 MBq, 1.5 min acquisition time per bed position, 30% overlap between bed positions). Q.Clear reconstruction was applied with 384 × 384 matrix resulting in 1.82 × 1.82 × 2.79 mm voxel size (Fig. 1 upper row). The local ethics committee approved the use of patient data in this study.

**Fig. 2:**
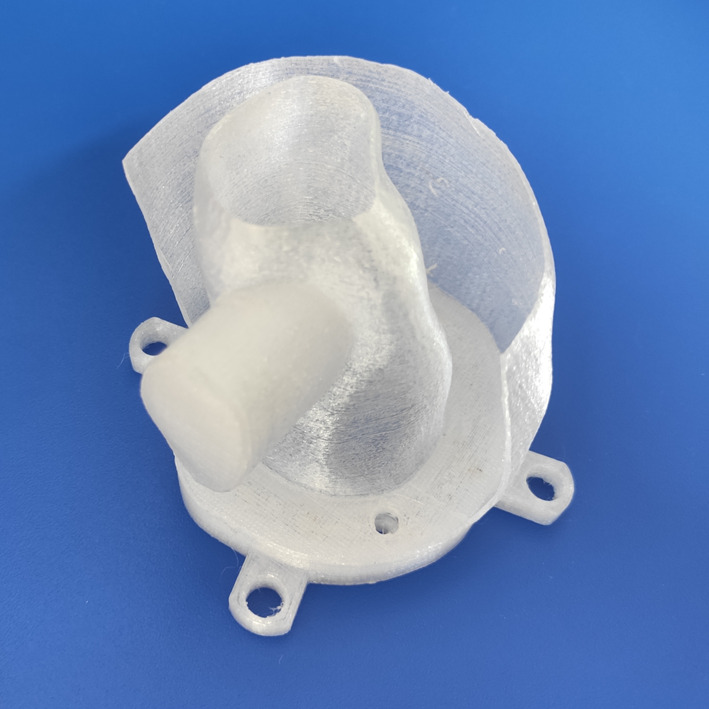
Multi-sectional image of the real 3D printed LVm defective phantom

For image segmentation process, we used the 3D Slicer software with basic functionality [30]. Images were cropped near to the area of the heart. Segmentation was done by applying the Otsu method with minimum and maximum threshold values obtained by visual inspection [31]. Irrelevant segmented voxels were deleted manually using the Erase tool. The 3D mask was saved in Standard Tessellation Language (STL) format with LPS (Left, Posterior, Superior) coordinate system and size scale of 1.0. The exported model was post-processing using Autodesk Meshmixer (Autodesk Inc., San Rafael, California, USA). The Plane Cut tool was used to make a plane surface on the left ventricle model from the direction of the left atrium perpendicular to the apex. A 0.5 mm offset distance was defined to create the model hollow, while solid accuracy and mesh density parameters were set to 512 in the Hollow tool. The size of the plain surface of the model was increased with the Extrude tool to make a 10 mm wide solid pedestal. In addition, with the Hollow and Extrude tools, a bubble trap was created. A phantom holder was also created in Trimble SketchUp Pro 2020 (Trimble Inc., Sunnyvale, California, USA) based on the distance and size of the pedestal holes of the commercially available Biodex Cardiac insert. This holder was merged with the previously created plane surface of the cardiac model in Meshmixer using Boolean Union method (Fig. 1). As a last step, two filling holes were designed, one of them through the bubble trap. Finally, with our primary purpose, two types of phantom models were designed: one as a representation of the healthy LVm with 190 ± 1 ml fillable volume (Fig. 1 lower row), and another mimicking transmural perfusion defective myocardium with a 20 × 30 mm oval solid plastic cold part (Fig. 2). The latter model has 165 ± 1 ml total fillable volume. These two phantoms will be referred to in the following as LVm healthy and LVm defective phantom.

### 3D printing

**Fig. 3:**
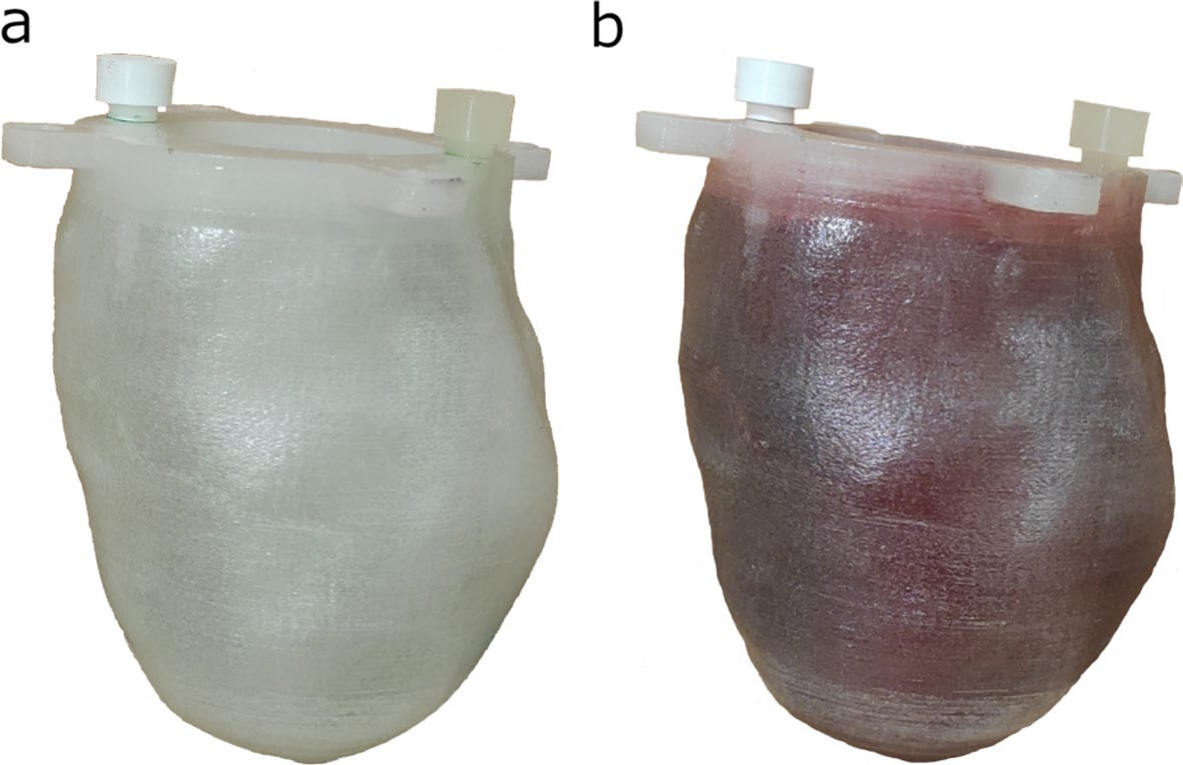
Photographs of the real 3D printed LVm healthy phantom before (**a**) and after (**b**) a red food-dye diluted water filling

For slicing and creating the print plan, the Repetier-Host (Hot-World GmbH & Co. KG, Willich, Germany) software was used. The model was laid flat on its pedestal. Slicing parameters were the followings: 100% infill density; shell thickness was 0.4 mm with 0.2 mm layer height. No adhesion or support was generated for the 40 mm/s print speed. Retraction and cooling were enabled. Phantoms were printed using an Anet A8 FDM 3D printer (Anet Technology Co., Ltd., Shenzhen, People's Republic of China), build volume 220 × 220 × 240 mm, Marlin firmware, 0.4 mm nozzle diameter, with 3DJAKE PETG transparent filament. PETG thermoplastic was used for watertight and durability reasons and to avoid significant stringing, which is a well-known phenomenon in the case of other printing materials (e.g., PLA) [32]. Print bed and nozzle temperature were set to the mid-value of the manufacturer's recommended temperature ranges: 70 °C and 240 °C, respectively. The total 3D printing time was approximately 6 h for each model. The 0.4 mm nozzle diameter and 0.4 mm shell thickness print parameter give 0.4 mm real wall thickness to the printed phantoms. A few times, the phantom had clearly visible separate layers on the outer apex side after printing, in these cases, we used a soldering iron to melt them together. To prevent any further leakage between layers, Prisma Color Acrylic spray was applied to the outer surface on the printed phantoms as a finishing process. To assure as less leaking as possible, M5 size screws were 3D printed to tightly fit in the phantom filling holes (Fig. 3).

**Fig. 4:**
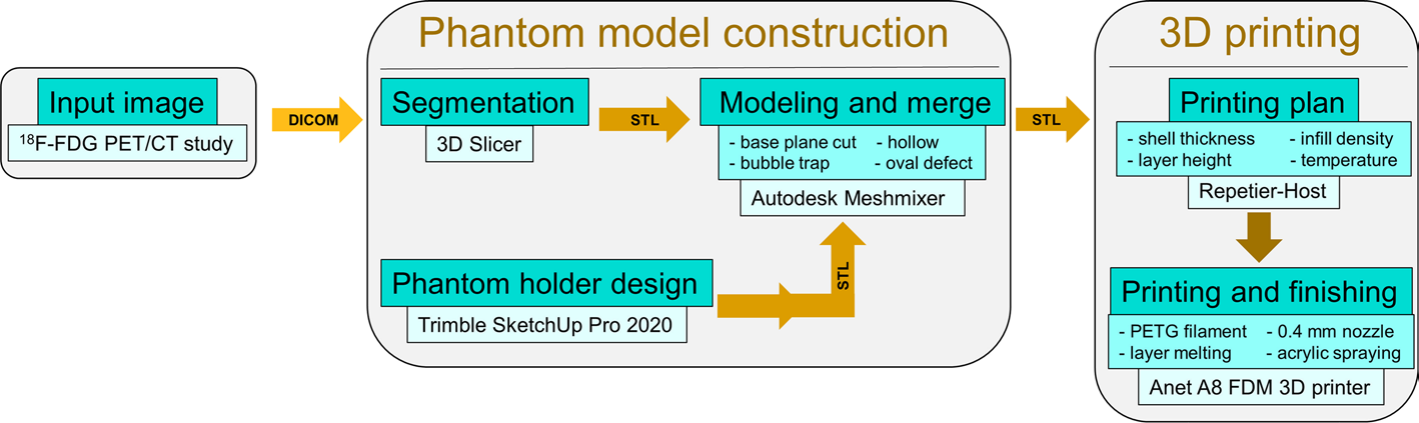
Flowchart of steps required to create the LVm phantom models. Input data were an ^18^F-FDG PET/CT study of a patient without known coronary artery disease. Three different software were used for model construction, and an additional program was applied to create the printing plan at different stages of the manufacturing process. Finished models were printed with an Anet A8 FDM 3D printer

The described steps required to create LVm phantom models are shown in Fig. 4. Our 3D phantom model is available in STL format in the supplementary material.

### Printing reproducibility, leaking test

Reproducibility of the phantom production was demonstrated with three separate printing series. Size, including the diameter of the pedestal and filling holes, was measured with a sliding calliper. Spiral CT scans with 120 kV, 120 mA x-ray settings, and voxel size of 0.625 × 0.703 × 0.703 mm were performed and evaluated on healthy phantoms filled with water to measure the accuracy of reproducibility. For the leakage test, watertight fillings were checked at least two times for each phantom (Fig. 3).

### Phantom SPECT/CT measurements

^99m^Tc- water solution was mixed with red food-dye for better visual detection of bubbles and leakage. Decay corrected activity concentrations calculated to the acquisition start can be seen in Table 1.

**Table 1:** Decay corrected activity concentrations in kBq/ml applied for phantom preparations

Measurement type	Volume [ml]	Activity concentrations (kBq/ml)		
		"in air"		"in torso”
Background	10100	–	–	20.15
Liver	1200	–	–	62.45
LVm healthy phantom	190	72.74	–	59.8
LVm defective phantom	165	–	69.04	–

In the first experiments, LVm healthy and defective phantoms were measured without additional scattering media ("in air”). The LVm healthy insert was also placed into the Anthropomorphic Torso Phantom for a second acquisition (“in torso”)

### Measurements and reconstructions

**Fig. 5:**
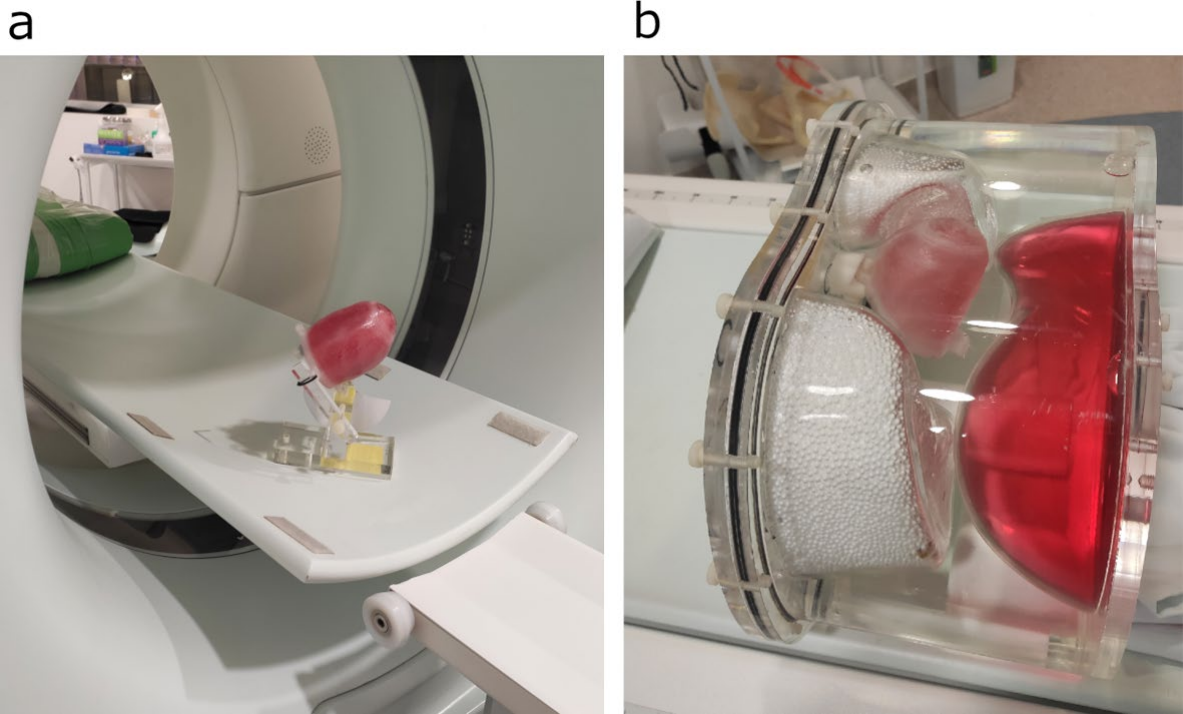
"In air” measurement positioning of LVm phantom on the SPECT/CT scanner bed (**a**), and the “in torso” setup with the Anthropomorphic Torso Phantom, when the LVm phantom is placed at the location of the original heart insert (**b**)

Both LVm healthy and LVm defective phantoms were measured without additional scattering media (referring to as "in air” measurement). The LVm healthy insert was placed into the Anthropomorphic Torso Phantom for a second acquisition (referring to as "in torso” measurement). All imaging acquisitions were performed with identical acquisition parameters on a NaI(Tl) detector-based AnyScan® DUO FLEX SPECT/CT system (Mediso Medical Imaging Systems, Budapest, Hungary) equipped with Low energy High-Resolution (LEHR) parallel hole collimator. The different phantom arrangements on the SPECT/CT scanner bed can be seen in Fig. 5. The routine clinical patient protocol for myocardial perfusion was selected, including the following parameters: 90 degrees scan arc, 64 projections, 128 × 128 matrix size, 140.5 keV energy with 20% window width, body contouring, and step and shoot mode. Additionally, a CT scan with 120 kV, 50 mA x-ray settings, and a voxel size of 2.50 × 0.977 × 0.977 mm was performed for attenuation correction purposes.

### Data processing

Data were processed by the Mediso InterView™ XP application. Default Cardiac Perfusion Image reconstruction of Tera-Tomo™ 3D SPECT-Q was applied on the acquired raw data. Image size of 128 × 128 with 5.91 mm cubic reconstruction voxel size, 32 number of iterations and 4 subset size was used with CT-based attenuation and scatter correction. Polar maps with 17 segments were created for all three measurements and were applied to reveal similarities and differences. For this process, reorientations were performed by a medical expert physician with six years of experience. Polar maps of "in air" measurements of the healthy and defective myocardium phantoms were compared, to demonstrate how the defect alters the internal distribution of the radioactive solution inside the phantom. Since the "in torso” measurement represents more realistic scattering and attenuation conditions, the polar map of the "in air" measurement of the healthy myocardium phantom was compared to the polar map of the "in torso” measurement. Polar map graphs from the calculated percentage differences were also created for these evaluations.

## Results

**Fig. 6:**
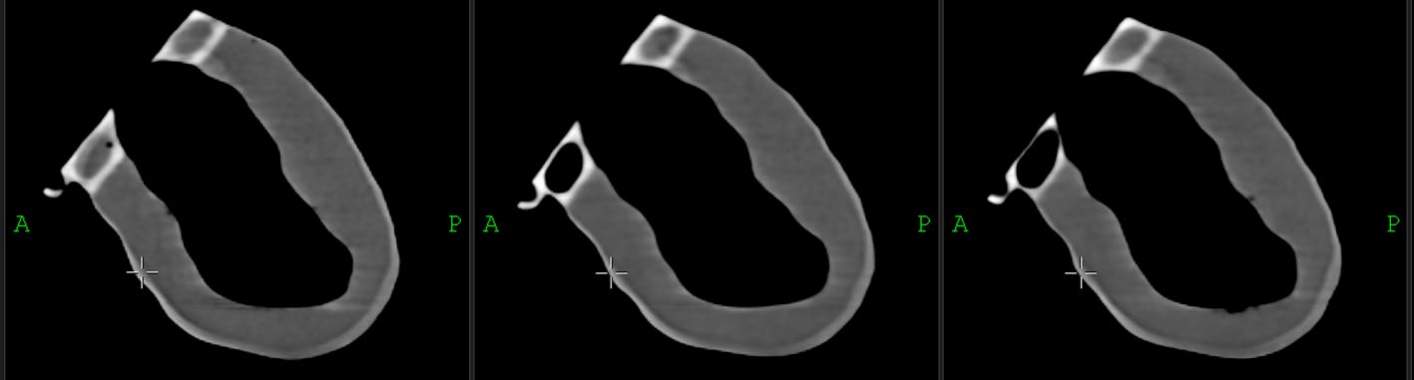
Representative sagittal view of the registered CT images of the three identical LVm healthy phantoms

After rigid registration of the high-resolution CT images of LVm healthy models, the phantoms present identical geometry within tight tolerances in shape (Fig. 6) and fillable volume.

**Fig. 7:**
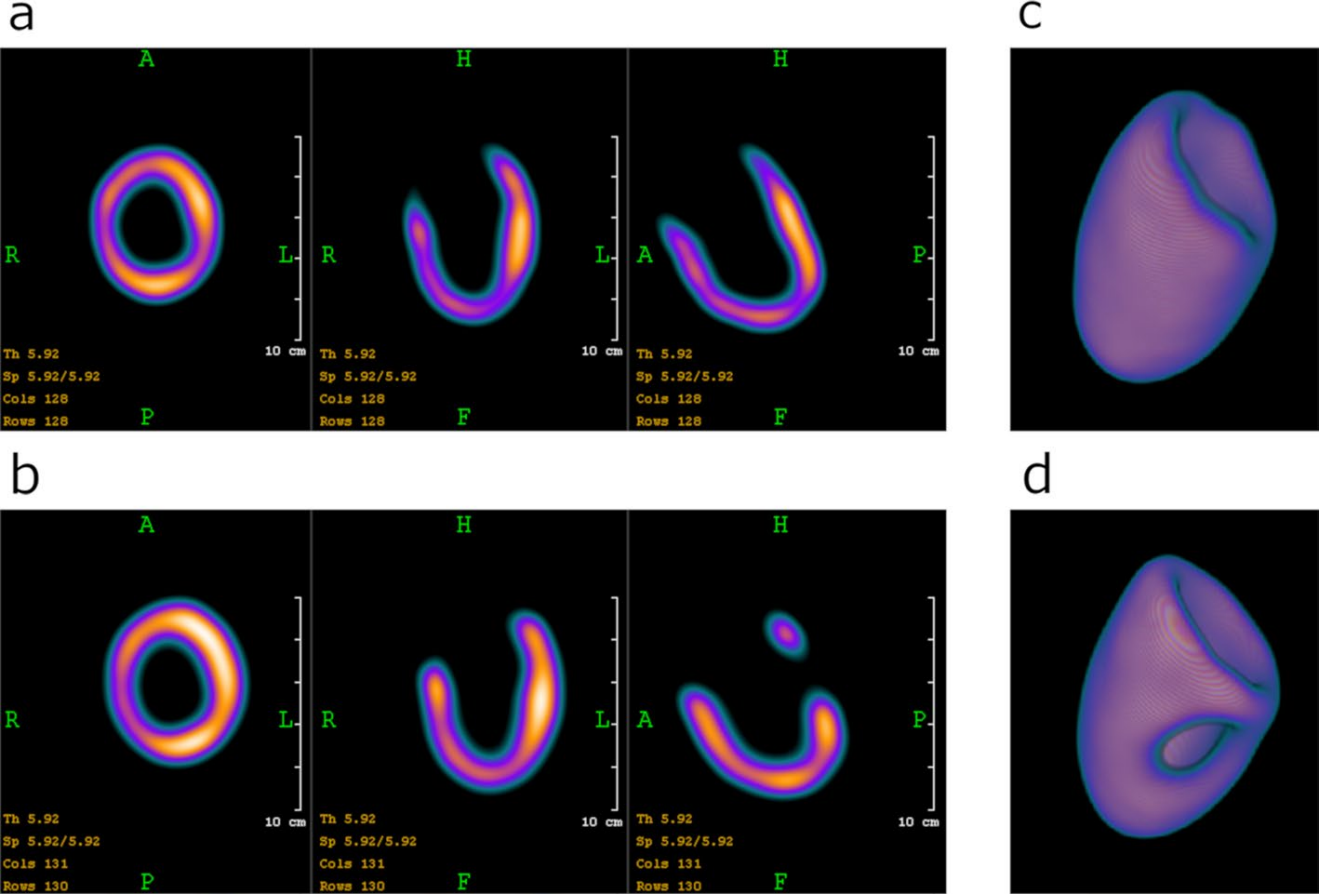
Uptake patterns of reconstructed SPECT images of LVm healthy and defective phantoms measured "in air” on three orthogonal views (**a**, **b**) and 3D rendered images of the two phantom realizations (**c**, **d**)

The measured mean filled volume was 189.4 ml ± 1.4 ml, including the volume of the bubble trap. The printed LVm phantoms were easily refillable and were closed tightly, without any air bubbles or observable leakage during all of the presented measurements. Additionally, two phantoms were filled and stored for three months at room temperature, and no leakage or evaporation was detected. Reconstructed SPECT images of "in air” measurements reveal accurate uptake volumes (Fig. 7). Differences between defective and healthy phantom images were clearly visible on the sagittal views (Fig. 7a, b) as well as on the 3D rendered image (Fig. 7c, d). The defect appeared where it was planned during the phantom design.

**Fig. 8:**
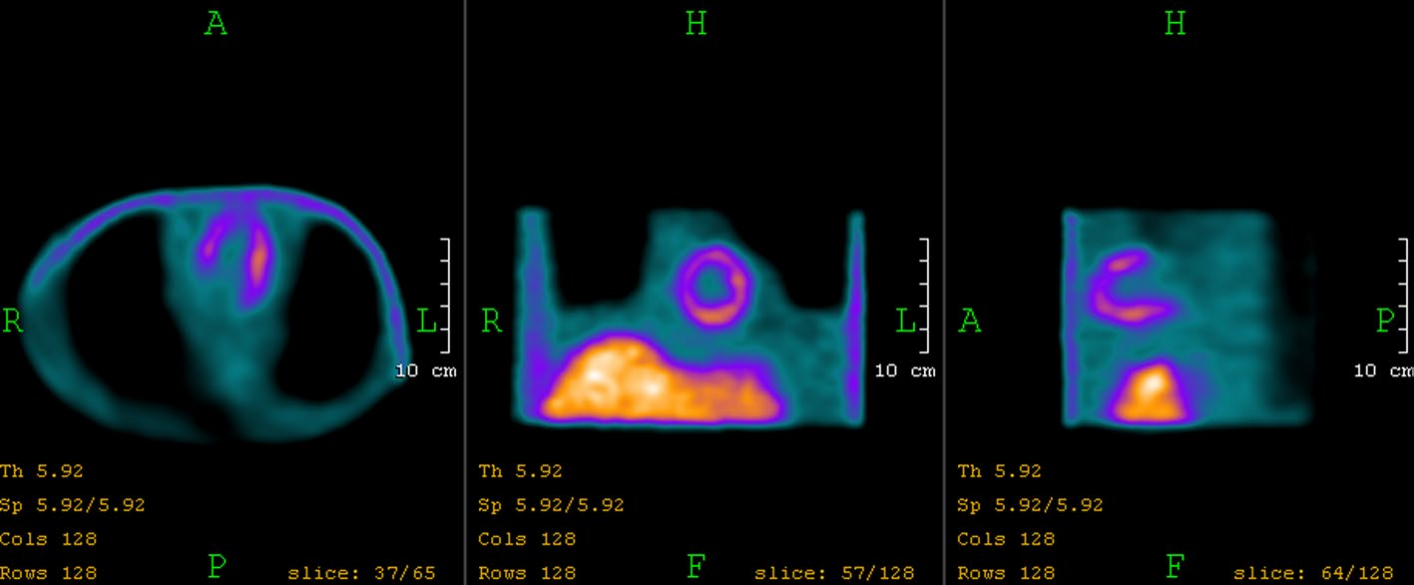
Reconstructed SPECT image of the LVm healthy phantom inserted in the Anthropomorphic Torso Phantom

Printed phantoms were compatible with the Anthropomorphic Torso Phantom to be assembled at the cardiac region. The reconstructed SPECT images revealed that the activity distribution of the LVm healthy phantom could be visualized in detail (Fig. 8).

**Fig. 9:**
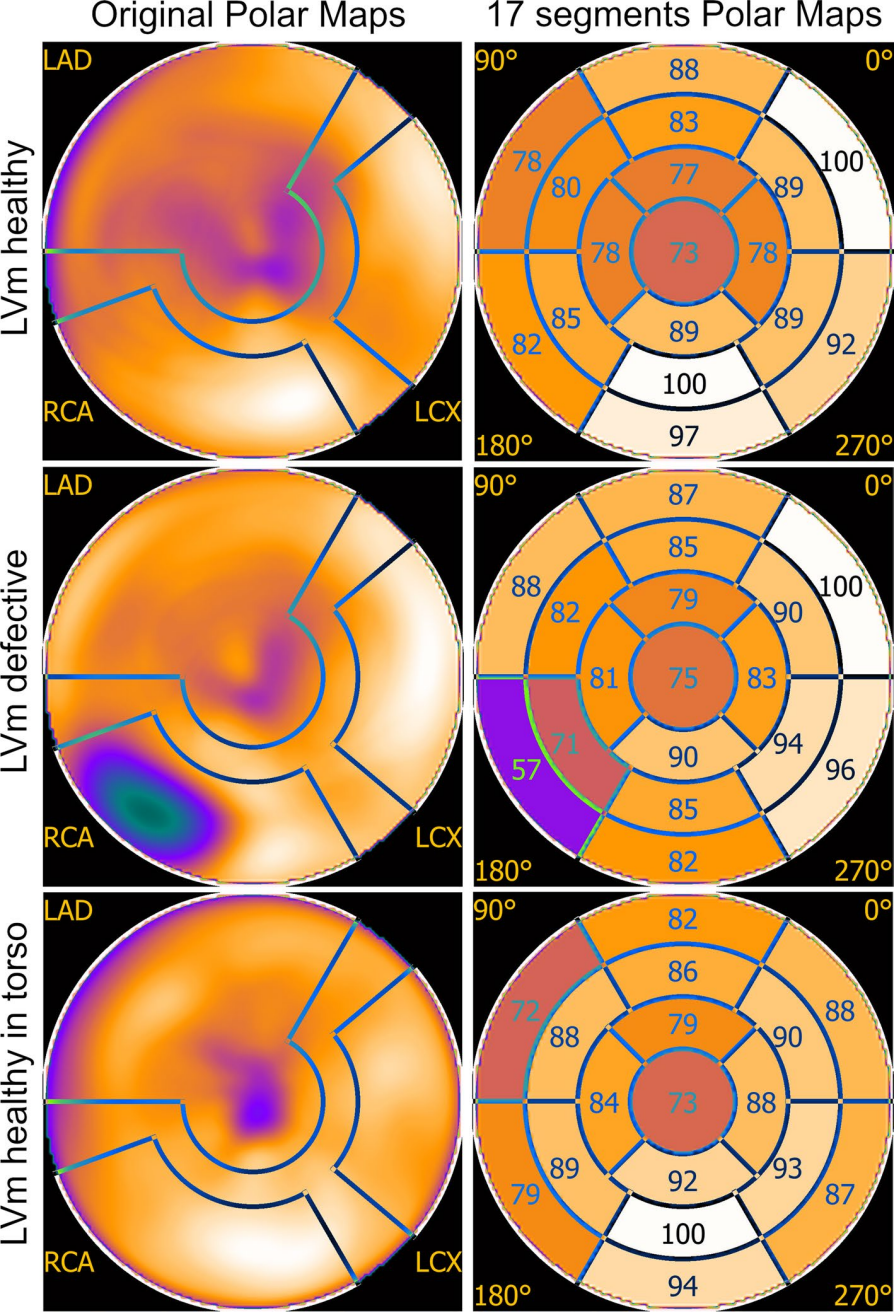
Original (first column) and 17 segments (second column) polar maps of the three measurements

Clear differences were found while analyzing the resulted polar maps of the three measurements of the "in air” and "in torso” arrangements (Fig. 9).

The polar map segment with the highest signal was found to be the basal anterolateral for LVm healthy and LVm defective "in air” measurements. On the other hand, the originally high signal mid-inferior region on the LVm healthy model was decreased significantly due to the artificial defect on the LVm defective model.

Polar map segment differences of the LVm healthy phantom measurements (first and last rows in Fig. 9) could originate from at least two sources. The radiopharmaceutical activity decayed compared to the "in air” case; therefore, the overall signal yield was expectedly lower. Moreover, the liver and the background in the torso phantom contained image distortions due to the spillover effect. All three polar maps have similar apex and apical region values.

The detailed relative perfusion percentage values of each region for all three measurements are summarized in Table 2, together with the relative percentage difference of measurement comparisons.

**Table 2:** Polar map values for each measurement and related percentage differences

Area	I. LVm healthy phantom "in air” (%)	II. LVm defective phantom "in air” (%)	III. LVm healthy phantom "in torso” (%)	IV. Relative % difference of I. and II	V. Relative % difference of I. and III
Basal anterior	87.5	87.3	82.2	− 0.2	− 5.3
Basal anteroseptal	77.6	88.2	72.4	10.6	− 5.2
Basal inferoseptal	82.0	57.3	79.4	− 24.7	− 2.6
Basal inferior	97.2	81.8	93.5	− 15.4	− 3.7
Basal inferolateral	92.0	95.9	86.6	3.9	− 5.4
Basal anterolateral	100.0	100.0	87.6	0.0	− 12.4
Mid-anterior	83.0	85.3	85.8	2.3	2.8
Mid-anteroseptal	79.7	81.5	88.4	1.8	8.7
Mid-inferoseptal	84.8	71.2	88.9	− 13.6	4.1
Mid-inferior	99.9	84.7	100.0	− 15.2	0.1
Mid-inferolateral	89.1	93.9	92.8	4.8	3.7
Mid-anterolateral	88.8	90.0	90.5	1.2	1.7
Apical anterior	76.9	78.6	79.5	1.7	2.6
Apical septal	77.6	81.1	84.0	3.5	6.4
Apical inferior	88.6	89.6	92.4	1.0	3.8
Apical lateral	78.3	83.4	88.5	5.1	10.2
Apex	73.1	75.0	73.2	1.9	0.1

The last two columns show the relative % difference for each segment between "in air” measurements (column I. and II.) and between the LVm healthy phantom "in air” and "in torso” measurements (column I. and III.)

**Fig. 10:**
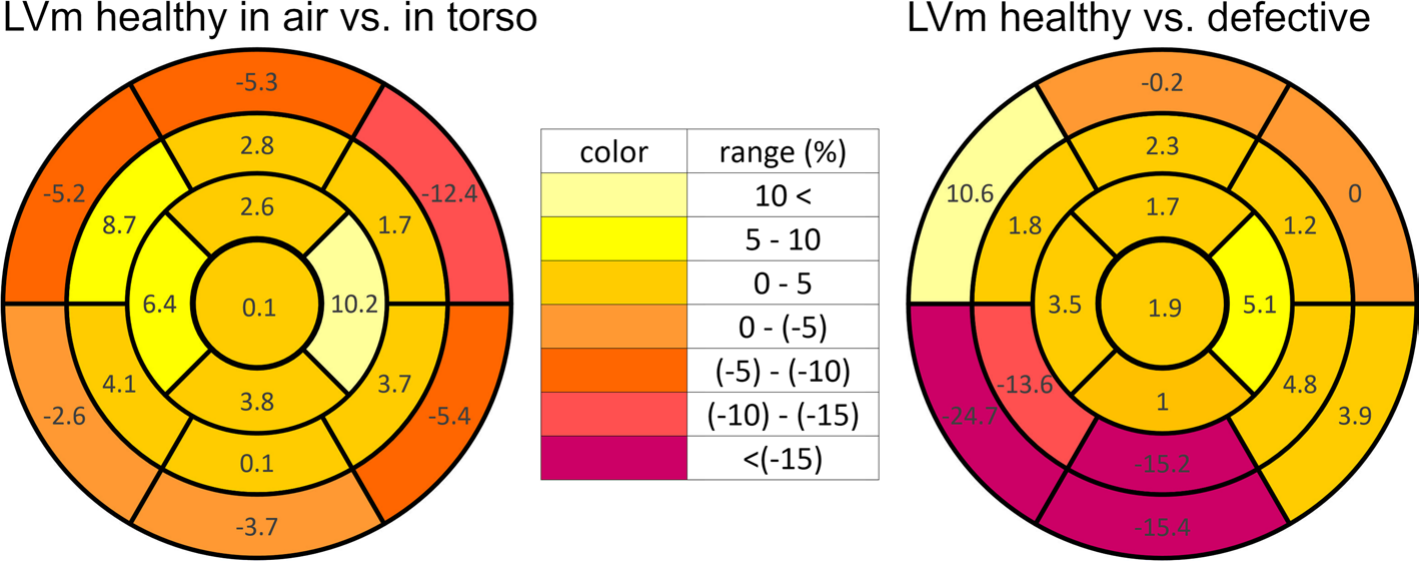
Polar maps of the relative percentage differences for different LVm phantom measurements. Left panel: results of the LVm healthy phantom "in air” vs. "in torso” comparison. Right panel: results of the LVm healthy vs. LVm defective phantom comparison

In the relative % difference columns (column IV. and V.), negative value means deterioration, while a positive represents an improved region. The values of the LVm healthy—LVm defective phantom comparison (column IV.), are in the range between − 24.7% and 10.6%, and 11 of the 17 segments have less than 5% value. The LVm healthy phantom "in air”–"in torso” comparison (column V.) has values between − 12.4%, and 10.2%, and 10 of the 17 segments have values less than 5%. The relative percentage differences were also depicted on differential polar maps (Fig. 10) based on column IV. and V. values of Table 2. Each color indicates 5 percentage steps. At the LVm healthy versus LVm defective phantom comparison, the deviations were higher than 5% deterioration concentrated on the four inferior regions where the artificial defect was designed. Two regions showed an improved signal ratio of more than 5%. Improvement and relapse regions in the case of LVm healthy phantom "in air” versus "in torso” comparison did not come from the nature of our phantom. As the concerning graph shows, the deviation is located in the basal edge regions, while the values are still around 5% except for the basal anterolateral region. This result and the 10.2% improvement in the apical region can be attributed to the uncertainty of the manual heart reorientation.

## Discussion

While several conventional plastic phantoms are available to test the image quality and reliability of nuclear cardiology applications with SPECT [8, 33–35], they still have some anatomical and size limitations. 3D printing technology has gained wide attention recently for creating anthropomorphic phantoms, due to its cost-effectiveness, fast production capability and the possibility for advanced and customized design in almost any shape even for nuclear cardiology applications [28, 29]. In this work, two anatomically accurate LV myocardial phantom inserts were created from a real patient ^18^F-FDG PET/CT study image set (Fig. 1 upper row). One represents the original healthy LV myocardium (Fig. 1 lower row), and the other includes an artificially added myocardium deficit (Fig. 2). Three LVm healthy phantoms were 3D printed to verify that there are no significant alterations in geometry (Fig. 6.) and fillable volume (189.4 ml ± 1.4 ml). These phantom inserts were planned to be convenient and complementary solutions to the commercially available plastic phantoms used in nuclear cardiology. Bubbles in the myocardium volume of the LV phantoms could affect the distribution of the radioactive solution. The LV insert of the Anthropomorphic Torso Phantom has no bubble trap, while the 3D printed LVm inserts were designed to include one for bubble-free filling of the LV wall. Therefore, the imaging of our phantoms was not affected by the presence of bubbles in the artificial myocardium volume. Moreover, the conventional LV insert is available at a certain size in a geometrically simple shape [33]; however, our 3D printed LVm insert is scalable in size and results in a more realistic uptake pattern of the LV myocardial perfusion SPECT image (Fig. 7). The latter has particularly high significance in the case of testing optimal settings for image reconstruction algorithms to avoid artifacts. The LV myocardium wall has a significantly different cross-sectional diameter at the apex than other regions, and the iterative image reconstruction tends to reach accurate activity levels at different iteration numbers for the apex than to the LV walls [36] even in case of a geometrically simple LV phantom. This is more prominent when we consider the real anatomy of the LV with non-uniform wall thickness. Therefore, our phantom design is a good advocate to the geometrically simple LV phantoms to find optimal iteration number for a certain image reconstruction. In addition, the reduction in left ventricular apical tracer uptake called apical thinning or false apical defect [37] is frequently observed in myocardial perfusion imaging both in the field of PET [38] and SPECT imaging [39, 40]. Among many potential causes, the diminished activity at the apex can be attributed to real anatomy [41] combined with the partial volume effect, as it is visible in our phantom model as well (Fig. 6). Another commercially available phantom called the Kyoto HL cardiac torso phantom (Kyoto Kagaku Co. Ltd., Kyoto, Japan) was used by Yoneyama et al. to test image reconstruction resolution recovery solutions to overcome ejection fraction (EF) limitations in case of pediatric patients [42]. However, with our method, two small size hearts can be printed from normal gated PET image sets in end-systole and end-diastole phases, and the EF measurement accuracy of different reconstruction methods can be tested [43]. It has to be mentioned that the commercially available AGATE phantom [8] can mimic simple heart motion and is compatible with the Anthropomorphic Torso Phantom. Therefore, gated SPECT acquisitions and EF calculation are possible; however, this phantom is also available only in adult patient size. The anatomically correct design of the LV myocardium is also important when comparing hybrid or ellipsoid sampling of polar map generation [44]. A set of printed phantoms with different clinically representative cases could be used to perform a comparison of existing nuclear cardiology software, since considerable differences are present in their performance [45]. We performed a representative set of SPECT image acquisitions using the 3D printed phantom inserts. Both LVm healthy and LVm perfusion defect phantoms were filled with ^99m^Tc, and SPECT acquisitions were performed on an AnyScan® DUO FLEX SPECT/CT system "in air”, without any scattering media and in the Anthropomorphic Torso Phantom including hot background and hot liver insert. The printed LVm models remained intact throughout the experiments, and the inserted ^99m^Tc radioactive solution did not dissolve into the torso phantom background chamber. Seventeen segments polar map analysis of SPECT images revealed that by comparing the LVm defective model to the LVm healthy one, a significant deficit in the radiopharmaceutical distribution appeared where it was expected (Fig. 9). The design process enables flexibility in placing the perfusion deficit with different numbers and shapes within the fillable wall of the phantom. Including the expected low perfusion segments at the deficit area, around 65% of the polar map segments had less than 5% deviation. When comparing the LVm healthy model, the "in torso” and "in air” measurements, 59% of all polar map segments had less than 5% deviation (Fig. 10). Concerning only the segments excluding the deficit area, neither comparison revealed more than 12.4% deviation, which difference could have originated most probably from phantom positioning error, the applied reconstruction method, and the well-known spill-over effect, especially in the case of the "in torso” phantom measurement. Beyond the flexibility and applicability of our method, this study has several limitations. We used an ^18^F-FDG PET image set to create the phantom model; however, a more realistic model can be created with currently available PET myocardial perfusion traces such as ^82^Rb-chloride, ^13^ N-ammonia or ^18^F-flurpiridaz. We presented 3D printed phantoms in one LV size from a non-gated PET data of a healthy male patient. It would be beneficial to demonstrate phantom studies using healthy females and pediatric or even heart disease images as input data. On the other hand, the variety of these printable LVm phantoms should be limited to provide a few standardized shapes available to be downloaded and printed in any nuclear cardiology laboratory. The LV deficit designed and printed in the phantom was completely solid, representing scar burden. However, a fillable defect could be printed within the LV wall, and lower activity concentration can be inserted in that chamber to mimic ischemia. In this study, only the LV was segmented; however, the anthropomorphic nature of the phantom could be emphasized with a model including the right ventricle as well.

## Conclusion

In this study, we proved that creating a fillable, anthropomorphic 3D printed phantom of the LV myocardium segmented from a real patient PET image volume is possible. SPECT images were acquired in different imaging scenarios proving the usefulness of the printed LVm phantoms. The flexibility of the 3D printing process presented in this study provides scalable and anthropomorphic image quality phantoms in nuclear cardiology imaging.

## Data Availability

Our phantom inserts are available in STL format in the supplementary material.

## Abbreviations

DLP: Digital light processing
EF: Ejection Fraction
FDM: Fused deposition modelling
LEHR: Low Energy High-Resolution
LPS: Left, Posterior, Superior
LV: Left ventricle
LVm: Left ventricle myocardium
PETG: Polyethylene terephthalate glycol
PLA: Polylactic acid
SLA: Stereo-lithography
STL: Standard Tessellation Language
